# Job satisfaction of the primary healthcare providers with expanded roles in the context of health service integration in rural China: a cross-sectional mixed methods study

**DOI:** 10.1186/s12960-019-0403-3

**Published:** 2019-09-02

**Authors:** Yinzi Jin, Haipeng Wang, Dan Wang, Beibei Yuan

**Affiliations:** 1 Department of Global Health, School of Public Health, Peking University, 38 Xue Yuan Road, Haidian District, Beijing, 100191 China; 2 School of Health Care Management, NHC Key Laboratory of Health Economics and Policy Research, Shandong University, Jinan, 250100 China; 3 China Center for Health Development Studies, Peking University, Box 505, 38 Xue Yuan Road, Haidian District, Beijing, 100191 China

**Keywords:** Expanded roles, Job satisfaction, Primary healthcare providers, Mixed methods

## Abstract

**Objective:**

Against the backdrop of integrating public health services and clinical services at primary healthcare (PHC) institutions, primary healthcare providers (PCPs) have taken on expanded roles. This posed a potential challenge to China as it may directly impact PCPs’ workload, income, and perceived work autonomy, thus affecting their job satisfaction. This study aimed to explore the association between the expanded roles and job satisfaction of the PCPs in township healthcare centers (THCs), the rural PHC institutions in China.

**Methods:**

A cross-sectional study using mixed methods was conducted in 47 THCs in China’s Shandong province. Based on a sample of 1146 PCPs, the association between the proportion of PCPs’ working time spent on public health services and PCPs’ self-reported job satisfaction was estimated using the logistic regression. Qualitative data were also collected and analyzed to explore the mechanism of how the expanded roles impacted PCPs’ job satisfaction.

**Results:**

One hundred eighty-four physicians and 146 nurses undertook increased work responsibilities, accounting for 15.91% and 12.61% of the total sample. For those spending 40–60%, 60–80%, and more than 80% of the working time providing public health services, the time spent on public health was negatively associated with job satisfaction, with the odds ratio being 0.199 [0.067–0.587], 0.083 [0.025–0.276], and 0.030 [0.007–0.130], respectively. Qualitative analysis illustrated that a majority of the PCPs with expanded roles were dissatisfied with their jobs due to the heavy workload, the mismatch between the income and the workload, and the low level of work autonomy. PCPs’ heavier work burden was mainly caused by the current public health service delivery policy and the separation of public health service delivery and regular clinical services delivery, a significant challenge undermining the efforts to better integrate public health services and clinical services at PHC institutions.

**Conclusion:**

The current policies of adding public health service delivery to the PHC system have negative impacts on PCPs’ job satisfaction through increased work responsibilities for PCPs, which have led to low work autonomy and the mismatch between the income and the workload. The fundamental reason lies in the fragmented incentives and external supervision for public health service delivery and clinical service delivery. Policy-makers should balance the development of clinic and public health departments at the institutional level and integrate their financing and supervision at the system level so as to strengthen the synergy of public health service provision and routine clinical service provision.

## Introduction

In China, primary healthcare providers (PCPs) refer to the physicians and nurses working in the state-owned primary health care (PHC) institutions which consist of township healthcare centers (THCs) and village clinics. The expanded roles of PCPs mean that the PCPs have been engaged in the delivery of both public health services (individual preventive services and population health interventions) and clinical services (diagnosis and treatment). In China and other developing countries, providers may end up assuming expanded roles as a result of specific health programs. For example, in the case of HIV prevention services, the PHC institutions have to expand their routine services to include the delivery of maternal and neonatal care. The integration of public health services and basic clinical services at the PHC level is an evidence-based practice that can improve service accessibility and boost financial and operational efficiency, thus promoting sustainability and enhancing the quality of health care through service continuity and a more person-centered approach [1]. However, emerging studies have indicated that efforts to add specific public health services to PHC delivery systems in low-resource settings have been hampered by a number of challenges, including severe financial constraints, inadequate training and supervision for primary healthcare providers (PCPs), and poor policy and organizational management [2].

After the launch of a series of health policies in China, PCPs have undertaken more responsibilities as public health services were added to clinical services. Before China’s health care system reform beginning in 2009, PCPs at THCs focused mainly on providing clinical services because they had to rely on drug mark-ups and service fees as a source of financing in the context of limited government funding and support on public health service provision [3]. As a result, the demand for clinical services was supplier-induced, giving rise to surging health expenditures [4] and fewer accesses to health care [5]. Since 2009, two major policies have been introduced. The first one is the zero mark-up policy [6] which aims to control the induced service provision and drug over-prescription by removing the drug mark-ups at THCs [7]. At the same time, local governments increased budgets to make up for THCs’ lost revenue from drugs, but in most cases, the financial support is dependent on the local government’s fiscal capacity and not enough to offset THCs’ reduced revenues [8].

Another crucial policy is “Equalization of Basic Public Health Services” (EBPH), which aims to deliver essential public health services to every citizen, and according to which, the government subsidizes the THCs based on the number of covered residents and on the provider performance in terms of the designated public health services [9]. It is similar to the vertical health programs launched by other low- and middle-income countries (LIMCs), which target specific services and earmark funds according to the delivery performance of those specific services. Under the administrative pressure to meet the performance targets of providing the designated public health services so as to get the subsidies, THCs have to meet the government’s requirements for the designated public health services, follow the service provision procedure, and be subject to regular supervisions by higher authorities. Since the introduction of EBPH policy, THCs have input more human, financial, and infrastructure resources into the delivery of the designated public health services, most of which are newly added work content. In response, PCPs at THCs including physicians, nurses, and public health workers (PHWs) [10] need to devote more working time to the public health services designated by EBPH. Meanwhile, they also have to deliver basic clinical services to fulfill the major function of THCs.

Under such a circumstance, physicians and nurses at THCs have taken on heavier workloads to provide both clinical services and the newly added public health services, undoubtedly affecting their job satisfaction. PCPs’ low job satisfaction, a long-standing issue, can result in high turnover [11] and low performance of health care delivery [12, 13], thereby jeopardizing the sustainable development of the health system [14]. The bulk of research that studied the PCPs at THCs revealed that the heavy workload, low income, poor competence, few opportunities for career development, and incomplete organizational management were the factors leading to low job satisfaction in China [15–21]. Some studies also explored the influences of health system reforms on the job satisfaction of PHWs at THCs [22–25] Theoretically speaking, any health system reform would directly influence the motivating factors listed above, or how the health organizations design their management, thus in turn affecting what health workers can get from their work and their job satisfaction. For example, several studies found that due to the broad scope of public health services and limited financial incentives when the EBPH policy was first implemented, the PCPs felt stressful and torn between the many competing demands for their time [26, 27], thus often complaining about the lack of trust and cooperation of community members.

We assumed that the enforcement of delivering public health services at the PHC level could result in a situation where physicians and nurses had to provide more health services and face more intensive performance assessments. As a result, PCPs at THCs would live with extra work responsibility, heavier workload, and potential changes in income and autonomy, all of which are the important influencing factors of job satisfaction. With these points in mind, this study adopted a mixed method to evaluate the effects of expanding China’s THCs’s job scope to include public health services by (1) quantitatively examining the job satisfaction of the PCPs with expanded roles and (2) quantitatively and qualitatively exploring if the expanded roles have impact on job satisfaction by influencing the PCPs’ workload, income, and work autonomy.

## Methods

### Study location and context

The study sites included all the THCs in the three representative counties of Shandong province. We selected Shandong province because it is one of the first provinces to implement EBPH policy and is home to the largest number of both registered physicians and PHWs in China [28]. Located in northeast China, Shandong is the second largest province in China with a population of 99.47 million and ranks the third among the 31 provinces/municipalities in mainland China with a GDP per capita of ¥68 049 ($10 078).

The random cluster sampling was applied to generate a sample of three counties (Shouguang, Huantai, and Yanggu), equally representative of the high-, middle- and low-level economic region in the province. All the THCs in these three counties were surveyed, with 16 from Shouguang, 13 from Huantai, and 18 from Yanggu. All the PCPs on duty on the investigation day were encouraged to participate in the structured questionnaire survey and a total of 1146 participants were recruited.

### Quantitative data

#### Methods of measurements

##### Job satisfaction

This study analyzed the overall job satisfaction of PCPs as outcome variable, which was measured by the participants’ responses to the survey question “Are you satisfied with your work?” The responses of “very satisfied” and “satisfied” were coded as satisfied, while the responses of “moderate,” “dissatisfied,” and “very dissatisfied” were coded as dissatisfied.

##### Expanded work role

The key independent variable was defined as the proportion of time the PCPs spend on public health services and measured by participants’ responses to the survey question “What percentages of time do you spend respectively on providing clinical services and public health services per workday?” The two self-reported percentages should add up to 100%.

Considering that PCPs with expanded roles were subject to potential changes in workload, income, and work autonomy, all of which may influence PCPs’ job satisfaction, we also analyzed these three job characteristics as independent variables: (i) *workload*, defined as hours worked per week (< 40, 40–56, ≥ 57); (ii) *income level*, defined as monthly income (< 2000, 2000–2999, 3000–3999, ≥ 4000 Chinese Yuan, CNY); (iii) *work autonomy*, defined as the perceived autonomy to structure the working schedule and measured by the participants’ responses (yes or no) to the dichotomous survey question “Do you have control over your work, such as the autonomy to structure your schedule?” The “yes” response indicated high work autonomy, while the “no” response suggested low work autonomy.

##### Control variables

Informed of the conceptual framework based on the Warr et al.’s intrinsic and extrinsic model [29] and empirical research [29–34], we controlled other intrinsic satisfaction factors (relationship with fellows, relationship with patients, ability, chance of promotion, on-the-job training, attention paid to suggestions you make) and extrinsic satisfaction factors (workplace, benefit, performance appraisal, institutional administration). In this study, the demographic and social-economic status (SES) covariates included the PCPs’ age, gender, marriage status, educational background, professional status, employment status, and job type.

#### Statistical analysis

**Fig. 1:**
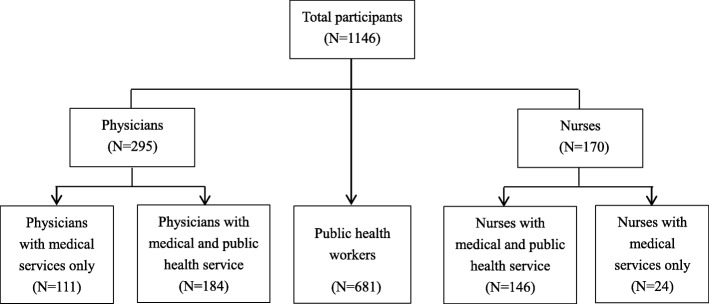
Flowchart of sample classification. This figure presents how the total participants were divided into five subgroups according to (1) the department where they are working and (2) whether they assume expanded roles

The total participants were divided into several subgroups according to the job types (Fig. 1). There were two steps of sample classification. First, we classified the PCPs into physicians, nurses, and PHWs according to the department where they were working (clinical departments or public health department). Second, in order to single out the PCPs with expanded roles, the PCPs working in clinical departments were further divided into four groups based on whether they undertook increased work responsibility: physicians providing clinical services only, nurses providing clinical services only, physicians with expanded roles, and nurses with expanded roles.

Descriptive statistics were used to describe the characteristics of the participants. Chi-square tests were conducted to determine the differences in socio-demographic characteristics of PCPs’ job satisfaction. To identify the association between the expanded roles and the PCPs’ job satisfaction, we fitted two multivariate logistic regression models (Appendix). In model 1, the job satisfaction was regressed on the proportion of the working time spent on public health. In model 2, additional regressors including workload, income, and work autonomy were added. Both models were adjusted for covariates, and the odds ratios (ORs) and their corresponding 95% confidence intervals (CI) were reported. The logistic regressions were also conducted on the four subgroups: physicians providing clinical services only, PHWs, physicians with expanded roles, and nurses with expanded roles. All statistical analyses were conducted using Stata 14.1 (StataCorp LP, College Station).

### Qualitative data

#### Data collection

We conducted face-to-face semi-structured interviews to better understand the expanded roles of PCPs and their influences on the job satisfaction from the perspectives of administrators and service providers. The interviewees were completely voluntary, and they were selected using purposive sampling after the questionnaire survey. Their gender, age, job type, and professional status were taken into consideration so as to make the opinions representative for all PCPs’ job satisfaction. Interviews were conducted in an individual meeting room without any non-participants for the privacy and comfort of the interviewees. Each interview lasted 30 to 60 min with on-spot notes and audio records taken. Interviews were conducted till the data saturation was reached.

The interviewees included 3 directors/section chiefs of County Health Bureau, 49 directors/vice-directors of THCs (14 from Shouguang, 16 from Huantai, and 19 from Yanggu), 46 physicians (16 from Shouguang, 12 from Huantai, and 18 from Yanggu), 64 PHWs (19 from Shouguang, 26 from Huantai, and 19 from Yanggu), and 40 nurses (15 from Shouguang, 9 from Huantai, and 16 from Yanggu). Three different interview outlines were designed respectively for the supervisors from County Health Bureau, the administrators of THCs, and the PCPs. The interview questions included how the implementation of national policies at the county level increased the public health service provisions at THCs, how THCs adjusted their institutional management, and how PCPs perceived their changed work contents, work autonomy, workload, and job satisfaction.

#### Data analysis

Theoretical coding was performed using NVivo 12.0. Two well-trained authors (YZJ, DW) were recruited to transcribe and code the audio records. Codes were revised and improved through reviewing transcripts and discussion. Finally, the coding framework was divided into the factors or practices affecting job satisfaction at the individual, institutional, and health system level, with each level being sub-coded by workload, autonomy, income, integration of public health services and clinical services, and other specific motivators.

## Results

### Quantitative study

As listed in Table 1, this study consisted of 1146 PCPs, with 15.91% being physicians with expanded roles and 12.61% being nurses with expanded roles. Most of the physicians and nurses with expanded roles were temporarily employed (59.24% and 76.03%). The physicians with expanded roles reported an average monthly income (3823.07 CNY) relatively lower than the total sample (4033.08 CNY) and a smaller proportion of them thought they had work autonomy (59.24%) than the total sample (62.47%).

**Table 1:** Characteristics of participants

Characteristics, *n* (%)	Overall	Physicians providing clinic services only	Public health workers	Physicians with expanded roles	Nurses with expanded roles	*P*
Gender						
Male	404 (35.28%)	44 (39.64%)	304 (44.70%)	19 (10.38%)	34 (23.45%)	< 0.00 1
Female	741 (64.72%)	67 (60.36%)	376 (55.29%)	164 (89.62%)	111 (76.55%)	
Age						
< 25	54 (4.73%)	4 (3.67%)	24 (3.53%)	18 (9.94%)	4 (2.74%)	< 0.00 1
25–34	335 (29.36%)	36 (33.03%)	180 (26.47%)	73 (40.33%)	41 (28.08%)	
35–44	546 (47.85%)	57 (52.29%)	337 (49.56%)	69 (38.12%)	75 (51.37%)	
45–54	157 (13.76%)	9 (8.26%)	104 (15.29%)	18 (9.94%)	20 (13.70%)	
≥ 55	49 (4.29%)	3 (2.76%)	35 (5.15%)	3 (1.66%)	6 (4.11%)	
Educational background						
Bachelor or above	438 (38.19%)	39 (35.13%)	275 (40.38%)	66 (35.87%)	52 (35.62%)	0.64 2
Junior college	481 (41.94%)	46 (41.44%)	272 (39.94%)	85 (46.19%)	64 (43.83%)	
High school or below	228 (19.88%)	26 (23.42%)	134 (19.67%)	33 (17.93%)	30 (20.55%)	
Professional status						
Senior/deputy senior	18 (1.58%)	1 (0.92%)	13 (1.92%)	3 (1.64%)	–	< 0.00 1
Intermediate	398 (34.88%)	22 (20.18%)	254 (37.46%)	61 (33.33%)	50 (34.25%)	
Primary	505 (44.26%)	51 (46.79%)	290 (42.77%)	83 (45.35%)	74 (50.68%)	
Lower than primary	220 (19.28%)	35 (32.11%)	121 (17.85%)	36 (19.67%)	22 (15.07%)	
Employment status						
Temporary	876 (76.17%)	79 (71.17%)	558 (81.94%)	109 (59.24%)	111 (76.03%)	< 0.00 1
Permanent	274 (23.83%)	32 (28.83%)	123 (18.06%)	75 (40.76%)	35 (23.97%)	
Hours worked per week						
Total	49.76 (9.73)	47.73 (7.43)	50.17 (10.03)	49.44 (9.93)	50.30 (10.02)	
< 40	26 (2.36%)	4 (3.74%)	10 (1.54%)	9 (5.06%)	2 (1.42%)	0.02 7
40–56	980 (89.01%)	100 (93.46%)	578 (88.79%)	153 (85.95%)	126 (89.36%)	
≥ 57	95 (8.63%)	3 (2.80%)	63 (9.68)	16 (8.99%)	13 (9.22%)	
Monthly income (CNY)						
Total	4033.08 (1463.78)	3747.66 (1245.55)	4103.79 (1465.87)	3823.07 (1517.57)	4189.14 (1473.90)	
< 3 000	285 (26.56%)	37 (36.63%)	160 (25.04%)	50 (28.41%)	31 (22.46%)	0.00 2
3 000–3 999	204 (19.01%)	16 (15.84%)	110 (17.21%)	43 (24.43%)	33 (23.91%)	
4 000–4 999	233 (21.71%)	29 (28.71%)	150 (23.47%)	29 (16.48%)	23 (16.67%)	
≥ 5 000	351 (32.71%)	19 (18.81%)	219 (34.27%)	54 (30.68%)	51 (36.96%)	
Work autonomy						
Feel high autonomy	714 (62.47%)	69 (62.16%)	433 (63.86%)	109 (59.24%)	89 (61.38%)	0.69 3
Feel low autonomy	429 (37.53%)	42 (37.84%)	245 (36.13%)	75 (40.76%)	56 (38.62%)	
Proportion of the working time spent on public health						
Total (%)	68.14 (41.68)	0	100	29.01 (23.98)	31.81 (25.35)	< 0.00 1
< 20	276 (24.08%)	111 (100%)	–	82 (44.56%)	59 (40.41%)	
20–40	81 (7.07%)	–	–	49 (26.63%)	32 (21.92%)	
40–60	52 (4.54%)	–	–	24 (13.04%)	28 (19.18%)	
60–80	29 (2.53%)	–	–	14 (7.61%)	15 (10.27%)	
> 80	708 (61.78%)	–	318 (100%)	15 (8.15%)	12 (8.22%)	
Job satisfaction						
Satisfied	875 (77.37%)	84 (75.67%)	519 (77.58%)	137 (74.86%)	113 (79.02%)	0.79 3
Dissatisfied	256 (22.63%)	27 (24.32%)	150 (22.42%)	46 (25.14%)	30 (20.98%)	

Table 2 presents the job characteristics of the PCPs with and without expanded roles. Compared with PCPs without expanded roles, a larger proportion of PCPs with expanded roles (those spend 60–80% and 80–100% of the working time providing public health services) worked more than 57 h per week (18.52% and 11.11% vs. 8.71%), and a lower proportion earned an income of ≥ 5000 CNY (20.69%, 15.38% vs. 32.16%) and reported lower autonomy (48.27%, 22.22% vs. 63.62%).

**Table 2:** Job characteristics of the PCPs without/with expanded roles

Job characteristics, *n* (%)	PCPs providing single clinic/public health services	PCPs spending 0–20% work time on public health services	PCPs spending 20–40% work time on public health services	PCPs spending 40–60% work time on public health services	PCPs spending 60–80% work time on public health services	PCPs spending 80–100% work time on public health services	*P*
Hours worked per week							
< 40	14 (1.85%)	2 (1.46%)	2 (2.53%)	3 (6.12%)	3 (11.11%)	1 (3.70%)	0.00 8
40–56	678 (89.45%)	119 (86.86%)	72 (91.14%)	46 (93.88%)	19 (70.37%)	23 (85.18%)	
≥ 57	66 (8.71%)	16 (11.68%)	5 (6.33%)	0	5 (18.52%)	3 (11.11%)	
Monthly income (CNY)							
< 3 000	197 (26.62%)	31 (23.48%)	16 (20.25%)	14 (29.17%)	10 (34.48%)	10 (38.46%)	0.00 1
3 000–3 999	126 (17.03%)	28 (21.21%)	32 (40.51%)	7 (14.58%)	6 (20.69%)	6 (23.08%)	
4 000–4 999	179 (24.19%)	21 (15.91%)	12 (15.19%)	9 (18.75%)	7 (24.14%)	6 (23.08%)	
≥ 5 000	238 (32.16%)	52 (39.39%)	19 (24.05%)	18 (37.50%)	6 (20.69%)	4 (15.38%)	
Work autonomy							
Feel high autonomy	502 (63.62%)	89 (63.57%)	59 (72.84%)	30 (57.69%)	14 (48.27%)	6 (22.22%)	< 0.00 1
Feel low autonomy	287 (36.38%)	51 (36.43%)	22 (27.16%)	22 (42.31%)	15 (51.72%)	21 (77.78%)	

Table 3 illustrates the association between socio-demographic/job characteristics and job satisfaction. Among the total 1146 participants, 91.32% of the PCPs with high work autonomy reported satisfaction, a percentage much higher than that of the PCPs with low work autonomy (54.25%). In the subgroup of physicians and nurses with expanded roles, those devoting more working time to public health felt more dissatisfied with their job (*p* < 0.001). For those with a monthly income of 2000 CNY or lower, only 58.00% of the physicians with expanded roles and 56.67% of the nurses with expanded roles felt satisfied with their job, while for those with a monthly income of more than 4000 CNY, the figures stood at 72.22% and 84.31%, respectively.

**Table 3:** Association between socio-demographic/job characteristics and PCPs’ job satisfaction

	Overall			Physicians with expanded roles			Nurses with expanded roles		
	Satisfied (%)	Dissatisfied (%)	*P*	Satisfied (%)	Dissatisfied (%)	*P*	Satisfied (%)	Dissatisfied (%)	*P*
Gender									
Male	296 (75.13%)	98 (24.87%)	0.20 1	17 (89.47%)	2 (10.53%)	0.11 8	24 (75.00%)	8 (25.00%)	0.54 2
Female	576 (78.47%)	158 (21.53%)		119 (73.01%)	44 (26.99%		88 (80.00%)	22 (20.00%)	
Age									
< 25	44 (84.62%)	8 (15.38%)	0.30 6	14 (77.78%)	4 (22.22%)	0.16 6	3 (100.00%)	–	0.63 1
25–34	258 (78.42%)	71 (21.58%)		49 (68.06%)	23 (31.94%)		31 (79.49%)	8 (20.51%)	
25–44	407 (75.37%)	133 (24.63%)		51 (73.91%)	18 (26.09%)		61 (81.33%)	14 (18.67%)	
45–54	124 (80.52%)	30 (19.48%)		17 (94.44%)	1 (5.56%)		14 (70.00%)	6 (30.00%)	
≥ 55	35 (71.43%)	14 (28.57%)		3 (100.00%)			4 (66.67%)	2 (33.33%)	
Educational background									
Bachelor and above	333 (77.08%)	99 (22.92%)	0.70 4	53 (80.30%)	13 (19.70%)	0.43 9	42 (80.77%)	10 (19.23%)	0.87 4
Junior college	371 (78.44%)	102 (21.56%)		60 (71.43%)	24 (28.57%)		49 (79.03%)	13 (20.97%)	
High school or below	171 (75.66%)	55 (24.34%)		24 (72.73%)	9 (27.27%)		22 (75.86%)	7 (24.14%)	
Professional status									
Senior/deputy senior	14 (77.78%)	4 (22.22%)	0.98 3	2 (66.67%)	1 (33.33%)	0.02 9			0.63 8
Intermediate	304 (77.16%)	90 (22.84%)		48 (78.69%)	13 (21.31%)		40 (80.00%)	10 (20.00%)	
Primary	386 (77.82%)	110 (22.18%)		66 (80.49%)	16 (19.51%)		55 (76.39%)	17 (23.61%)	
Lower than primary	166 (76.50%)	51 (22.50%)		20 (55.56%)	16 (44.44%)		18 (85.71%)	3 (14.29%)	
Employment status									
Temporary	663 (77.09%)	197 (22.91%)	0.69 7	83 (76.85%)	25 (23.15%)	0.45 7	83 (75.45%)	27 (24.55%)	0.05 6
Permanent	212 (78.23%)	59 (21.77%)		54 (72.00%)	21 (28.00%)		30 (90.91%)	3 (9.09%)	
Hours worked per week									
< 40	15 (57.69%)	11 (42.31%)	0.00 7	5 (55.56%)	4 (44.44%)	0.25 7	1 (50.00%)	1 (50.00%)	0.60 0
40–56	762 (78.64%)	207 (21.36%)		118 (77.63%)	34 (22.37%)		99 (79.20%)	26 (20.80%)	
≥ 57	66 (69.47%)	29 (30.53%)		11 (68.75%)	5 (31.25%)		10 (76.92%)	3 (23.08%)	
Monthly income (CNY)									
< 2 000	204 (72.60%)	77 (27.40%)	0.22 3	29 (58.00%)	21 (42.00%)	0.00 8	17 (56.67%)	13 (43.33%)	0.01 1
2 000–2 999	159 (80.30%)	39 (19.70%)		36 (85.71%)	6 (14.29%)		27 (87.10%)	4 (12.90%)	
3 000–3 999	176 (76.52%)	54 (23.48%)		25 (86.21%)	4 (13.79%)		19 (82.61%)	4 (17.39%)	
≥ 4 000	272 (77.94%)	77 (22.06%)		39 (72.22%)	15 (27.78%)		43 (84.31%)	8 (15.69%)	
Work autonomy									
Feel high autonomy	642 (91.32%)	61 (8.68%)	< 0.00 1	99 (90.83%)	10 (9.17%)	< 0.00 1	77 (89.53%)	9 (10.47%)	< 0.00 1
Feel low autonomy	230 (54.25%)	194 (45.75%)		38 (51.35%)	36 (48.65%)		35 (62.50%)	21 (37.50%)	
Proportion of the working time spent on public health									
< 20	230 (84.25%)	43 (15.75%)	< 0.00 1	74 (91.36%)	7 (8.64%)	< 0.00 1	51 (89.47%)	6 (10.53%)	< 0.00 1
20–40	72 (88.89%)	9 (11.11%)		41 (83.67%)	8 (16.33%)		31 (96.88)	1 (3.13%)	
40–60	36 (69.23%)	16 (30.77%)		14 (58.33%)	10 (41.67%)		22 (78.57%)	6 (21.43%)	
60–80	12 (41.38%)	17 (58.62%)		5 (35.71%)	9 (64.29%)		7 (46.67%)	8 (53.33%)	
> 80	524 (75.40%)	171 (24.60%)		3 (20.00%)	12 (80.00%)		2 (18.18%)	9 (81.82%)	

Table 4 shows the results of the logistic regression of the total sample and the two subgroups (physicians with expanded roles and nurses with expanded roles). Without adding the workload, income, and autonomy (model 1), the proportion of working time spent on public health (40–60%; 60–80%; more than 80%) were negatively associated with job satisfaction (OR = 0.199, *p* = 0.003; OR = 0.083, *p* < 0.001; OR = 0.030, *p* < 0.001). After adding the three job characteristics (model 2), for those who spent 60–80% and more than 80% of the working time on public health, the impact of the working time on job satisfaction decreased (OR = 0.036, *p* < 0.001; OR = 0.086, *p* < 0.001) and the negative associations had larger coefficient sizes. This could be interpreted that expanded roles influence job satisfaction of PCPs through workload, income, and work autonomy.

**Table 4:** Results of logistic regression on association between expanded roles and job satisfaction

		Model 1			Model 2	
	OR	95% CI	*P*	OR	95% CI	*P*
Overall						
Proportion of the working time spent on public health (reference, < 20)						
20–40	0.70 2	[0.24 6, 2.00 5]	0.50 9	0.74 3	[0.24 5, 2.25 8]	0.60 1
40–60	0.19 9	[0.06 7, 0.58 7]	0.00 3	0.16 5	[0.05 2, 0.52 1]	0.00 2
60–80	0.08 3	[0.02 5, 0.27 6]	< 0.00 1	0.08 6	[0.02 3, 0.31 4]	< 0.00 1
80–100	0.03 0	[0.00 7, 0.13 0]	< 0.00 1	0.03 6	[0.00 8, 0.15 9]	< 0.00 1
Work autonomy (reference, low autonomy)						
High autonomy				2.48 2	[1.55 4, 3.96 2]	< 0.00 1
Monthly income (reference, < 3 000)						
3 000–3 999				1.87 0	[0.94 8, 3.68 5]	0.07 1
4 000–4 999				2.71 8	[1.33 5, 5.53 3]	0.00 6
≥ 5 000				2.76 5	[1.32 7, 5.76 3]	0.00 7
Hours worked per week (reference, < 40)						
40–56				1.39 5	[0.41 0, 4.74 7]	0.59 4
≥ 57				1.17 8	[0.30 6, 4.53 0]	0.81 1
Physicians with expanded roles						
Proportion of the working time spent on public health (reference, < 20)						
20–40	0.38 9	[0.11 9, 1.27 0]	0.11 8	0.24 7	[0.05 4, 1.13 5]	0.07 2
40–60	0.11 5	[0.03 1, 0.42 4]	0.00 1	0.06 9	[0.01 2,0.41 1]	0.00 3
60 80	0.03 6	[0.00 7, 0.17 6]	< 0.00 1	0.02 6	[0.00 1, 0.50 9]	0.01 6
80–100	0.00 6	[0.00 3, 0.50 9]	< 0.00 1	0.02 1	[0.00 1, 0.41 6]	0.01 1
Work autonomy (reference, low autonomy)						
High autonomy				2.69 4	[1.67 1, 4.34 5]	< 0.00 1
Monthly income (reference, < 3 000)						
3 000–3 999				4.34 4	[1.55 0, 12.17 9]	0.00 5
4 000–4 999				4.52 6	[1.36 9, 14.96 0]	0.01 3
≥ 5 000				1.88 3	[0.83 0, 4.26 8]	0.13 0
Hours worked per week (reference, < 40)						
40–56				2.05 8	[0.18 8, 22.50 2]	0.55 4
≥ 57				1.35 9	[0.07 1, 25.95 7]	0.83 9
Nurses with expanded roles						
Proportion of the working time spent on public health (reference, < 20)						
20–40	3.93 8	[0.42 4, 36.59 8]	0.22 8	4.22 1	[0.34 4, 51.74 0]	0.26 0
40–60	0.41 6	[0.10 0, 1.72 3]	0.22 6	0.45 6	[0.08 7, 2.38 7]	0.35 2
60–80	0.10 4	[0.02 4, 0.45 2]	0.00 3	0.20 8	[0.03 6, 1.20 7]	0.00 8
80–100	0.02 2	[0.00 3, 0.15 2]	< 0.00 1	0.03 0	[0.00 3, 0.28 7]	0.00 2
Work autonomy (reference, low autonomy)						
High autonomy				1.94 9	[0.51 3, 7.39 5]	0.32 7
Monthly income (reference, < 3 000)						
3 000–3 999				5.16 2	[1.44 3, 18.46 1]	0.01 2
4 000–4 999				3.63 2	[0.99 2, 13.29 7]	0.05 1
≥ 5 000				4.11 0	[1.44 6, 11.68 1]	0.00 8
Hours worked per week (reference, < 40)						
40–56				0.60 0	[0.01 7, 21.66 7]	0.78 0
≥ 57				0.85 2	[0.01 4, 49.76 4]	0.93 9

As for the physicians with expanded roles, those devoting 40–60%, 60–80%, and 80–100% of the working time to public health were 93.1% (OR = 0.069, *p* = 0.003), 97.4% (OR = 0.026, *p* = 0.016), and 97.9% (OR = 0.021, *p* = 0.011) less likely to be satisfied with their job than those spending less than 20% of the working time providing public health. In the case of nurses with expanded roles, the negative associations between time spent on public health (40–60%, 60–80%, and 80–100% of the working time) and job satisfaction were weaker, respectively. Moreover, income level and work autonomy were positively associated with job satisfaction in the two subgroups.

### Perceptions of the relationship between expanded roles and job satisfaction

A majority of the PCPs with expanded roles expressed their dissatisfaction because ever since their routine job of providing clinical services at THCs was expanded to incorporate public health provision, they took on the heavy workload that was not compensated by higher income. In addition, they were not given enough autonomy.

Since the national health system reform in 2009, we need to spend large amount of working time providing the public health designated by EBPH. The performance appraisals are too frequent: quarterly, semi-annually and annually. We are struggling to cope with the assessments. It takes a lot of time and energy. (Physicians from THC in Yanggu)

The public health subsidy accounts for merely 20% of my total income. The proportion of performance-based bonus is even lower. I gain little from providing public health services, so I find it hard to be enthusiastic with my work. (Physician from THC in Shouguang)

I don’t think the residents can benefit a lot from the preventive health services, even though the effects cannot be identified in a short time. The assessment requirement and criteria of the providing public health services are too strict. We have to follow the work procedure and fill many forms, and there is no room for work autonomy. Another problem is that many residents do not appreciate our work and sometimes they feel bothered by the home visits. (Nurses from THC in Huantai)

### Perceptions of how expanded roles influenced job satisfaction

The interviews with administrators from County Health Bureau and THCs revealed how the EBPH policy affected PCPs’ job satisfaction through its impacts on external supervision and internal management of THCs, which then in turn influenced PCPs.

Physicians could get more income from clinical services provision, but could not get extra bonus for their input in public health work. The imbalance between inputs and rewards is a major source of dissatisfaction. This situation is more severe for the temporarily employed PCPs, whose monthly salary is ¥3000 lower than that of their permanently employed counterparts. In addition, the percentage of performance-based bonus in the total income was quite low. According to the assessment, the highest monthly income is only ¥200 to ¥500 higher than the lowest monthly income. (Director of THC in Yanggu)

The EBPH services provision performance are supervised and paid by County Health Bureau, while the clinical services provision at THCs are funded and supervised by Basic Health Insurance Schemes. The financing and delivery of clinical and public health services are fragmented. Measures have been taken to link the subsidy with performance, and to carry out intensive supervisions for the purpose of ensuring the universal and equal coverage of these basic services, but these measures have led to huge burdens for the THCs and PCPs working there. (Director of Huantai County Health Bureau)

Another challenge we face is the fragmented management of clinical and public health work within THCs. Clinical department and public health department usually have different functions and responsibilities, and are accountable to different supervisors. However, due to the expanded EBPH service package and a lack of qualified health professions in public health department, the public health services have to be allocated to some physicians or nurses. The combination of extra workloads and the difficulties in incorporating clinical services to the existing service provision is exerting pressures on physicians and nurses at THCs. (Director of THC in Shouguang)

## Discussion

This study adopted a mixed-method approach, using quantitative data to examine the relationship between expanded roles and job satisfaction and qualitative data to illustrate how the expanded roles influenced PCPs’ job satisfaction after the national health policy imposed public health service provisions upon THCs. To our knowledge, this study was the first to explore how the expanded role of providing both clinical and public health services affected PCPs’ job satisfaction of PCPs under China’s current health system reform. The EBPH public health service package and its segmented implementation in PCPs’ regular clinical work are the major reasons behind increased work burden for PCPs, which poses a significant challenge for integrating public health services into primary care institutions.

Several key findings were highlighted. First, this study confirmed the influences of workload, income, and work autonomy on PCPs’ job satisfaction. The overall satisfaction level of this sample (77.4%) was considerable with that of the American primary care physicians (80%) [22, 35] and that reported by PCPs in Chinese sample regions (70–80%) [16, 17]. Consistent with previous studies, we found that PCPs with higher income, lower workload, and higher work autonomy were more likely to be satisfied with their jobs [17, 18, 23, 24, 33]. Our findings provided suggestive evidence that the three job characteristics became important predictors of PCPs’ job satisfaction at the backdrop of China’s current health system reform.

Second, PCPs did take on expanded roles after public health service provisions were added to clinical services provision. PCPs’ increased work responsibilities were associated with their low job satisfactions, especially among the physicians with expanded roles. Although PCPs with expanded roles reported higher monthly incomes than PHWs, they had lower levels of satisfaction. There were two explanations. First, the income gap between PCPs with expanded roles and PHWs was not large enough to make up for PCPs’ additional inputs in public health; hence, the PCPs were demotivated. This was more prominent for physicians whose incomes were more related to their clinical services. Second, except for financial incentives, PCPs also took into account workload, autonomy, and other factors of job characteristics related to individual well-being and professional value. In particular, physicians value techniques of disease treatment and work autonomy more than other health professionals, and some physicians value clinical treatment services more than disease prevention services [5, 36]. Our results are in accordance with a number of studies’ findings that physicians in China were less satisfied with their job after the EBPH policy imposed public health services upon them [15, 16]. But few of the studies have further measured the impact of the expanded roles and provided in-depth explanations of the phenomenon. This study qualitatively probed the mechanisms behind the relationship: EBPH policy influences how THCs allocate work tasks and pay and supervise PCPs, resulting in a mismatch between workload and income level, and PCPs’ low level of work autonomy, both of which are closely related to their job satisfaction.

One explanation of the mechanism is that the expanded roles influenced the PCPs’ job satisfaction through the mismatch between income and workload. The median annual income of PCPs was about ¥ 48 000 ($6969), consistent with the data from previous studies [13] and lower than the China’s national average income (¥ 62 029, or $9000) [28]. The EBPH policy introduced in 2009 expanded the coverage of public health services, for which different levels of governments allocated funds. But according to the EBPH policy, the earmarked funds could be used only to support the operation costs such as material consumption and transportation, and not to compensate the personnel expenses. Consequently, PCPs’ input in public health provisions and increased workload cannot be rewarded enough. The explanation of the phenomenon is consistent with the previous studies that identified the unwillingness of health workers to deliver public health services due to concerns about rapidly increasing workload without corresponding financial rewards [2, 37, 38].

Another explanation is that expanded roles influenced PCPs’ job satisfaction through lower levels of autonomy. For physicians, public health services constitute extra workloads that are not connected with their routine clinical work. Therefore, providing public health services means more working time and less leisure time. Under the administrative pressure to meet the performance goals of public health service provision and in order to get the performance-based subsidies, THCs have to follow the government’s requirements for the designated public health services and procedures of service provision and accept regular assessments from different levels of supervisors, thus losing their work autonomy. Although a number of studies highlighted ongoing supervision as a predictor of the good performance of programs incorporating specific public health service to PHC delivery system [37, 39], this study indicates that as to how and how often the supervisions should be conducted to ensure the effectiveness, PCP’s perceptions of work autonomy should be given full consideration.

The qualitative interviews further revealed the fundamental reason of the fact that expanded roles negatively influenced PCPs who, however, in most cases, take providing both clinical and public health services simultaneously as granted. This discrepancy may be accounted for by the segmented financing and supervision for the clinical and public health services at the system level. Besides, this can also be attributed to a lack of integration of clinical and public health departments within THCs. In China’s PHC institutions, the communication and cooperation between clinical departments and the public health departments are scarce. Without integrating these two kinds of services, the delivery of public health services is simply perceived as extra work, an addition to the work burden. The findings are relevant for policymakers who should consider the following measures: strengthen the cooperation between clinical and public health departments at the institutional level and re-organize the financing and supervision of these two kinds of services at the system level in order to integrate clinical and public health services in PHC delivery system. Moreover, for other developing countries that are integrating vertical disease programs into primary care system, this study also presented the empirical experience that integration policies should take into account every critical function of the health system, including governance, planning, financing, monitoring, and service delivery.

This study has several limitations. First, our analysis was cross-sectional, limiting the interpretation of causal inferences. Second, the expanded roles were quantitatively measured by self-reported data. This self-administrated bias might have an impact on the results. However, the qualitative results provided in-depth explanation and served as a complement to quantitative results. Third, as only one province was selected in the sampling, the national representativeness of the study sample cannot be ascertained. The selected province is one of the first provinces to implement the policy of adding public health services package to the PHC institutions and is home to the largest number of both registered physicians and PHWs in China. Therefore, we believe that the selected counties are generally representative of the health and economic development in northeast rural China.

## Conclusions

This study found that PCPs did take on expanded roles as a result of the national policy requiring public health service provisions be added to the routine clinical service provision and the increased work responsibilities were associated with low job satisfaction of PCPs, especially among the physicians with expanded roles. The expanded roles might influence the PCPs’ job satisfaction through a mismatch between their workload and income, and a lower level of autonomy. Mixed methods were employed to enhance the generalization of the study findings. The fundamental reason of the phenomenon was the fragmented incentives, external supervision, and institutional administration for simultaneously delivering both the newly added public health services and the existing clinical services at PHC level. The findings can provide implication for policymakers to figure out better ways to coordinate the clinical and public health departments at the institutional level and to reform the financing and supervision at the system level. Moreover, for other developing countries that are integrating fragmented vertical programs at primary care level, this study provided an empirical experience that the integration policies cannot neglect any function of the health system, including governance, financing, monitoring, and service delivery.

## Data Availability

The data used and/or analyzed during the study are available from the corresponding author on reasonable request.

## Appendix

**Table 5:** Results of logistic regression on association between expanded roles and job satisfaction

		Model 1			Model 2	
	OR	95% CI	*P*	OR	95% CI	*P*
Overall						
Proportion of working time on public health (reference, < 20)						
20–40	0.70 2	[0.24 6, 2.00 5]	0.50 9	0.50 5	[0.15 2, 1.67 8]	0.26 5
40–60	0.19 9	[0.06 7, 0.58 7]	0.00 3	0.14 6	[0.04 2, 0.50 8]	0.00 2
60–80	0.08 3	[0.02 5, 0.27 6]	< 0.00 1	0.07 1	[0.01 6, 0.30 9]	< 0.00 1
80–100	0.03 0	[0.00 7, 0.13 0]	< 0.00 1	0.03 8	[0.00 8, 0.18 5]	< 0.00 1
Work autonomy (reference, low autonomy)						
High autonomy				2.56 0	[1.25 8, 5.21 1]	0.01 0
Monthly income (reference, < 3 000)						
3 000–3 999				2.11 6	[1.09 4, 4.09 2]	0.02 6
4 000–4 999				2.54 4	[1.27 3, 5.08 7]	0.00 8
≥ 5 000				2.56 0	[1.25 8, 5.21 1]	0.01 0
Hours worked per week (reference, < 40)						
40–56				2.14 1	[0.63 1, 7.26 8]	0.22 2
≥ 57				1.88 0	[0.49 2, 7.17 9]	0.35 5
Physicians with expanded roles						
Proportion of working time on public health (reference, < 20)						
20–40	0.38 9	[0.11 9, 1.27 0]	0.11 8	0.36 8	[0.10 3, 1.31 3]	0.12 3
40–60	0.11 5	[0.03 1, 0.42 4]	0.00 1	0.10 6	[0.02 3,0.47 3]	0.00 3
60–80	0.03 6	[0.00 7, 0.17 6]	< 0.00 1	0.01 7	[0.00 2, 0.13 0]	< 0.00 1
80–100	0.00 6	[0.00 3, 0.50 9]	< 0.00 1	0.03 5	[0.00 6, 0.19 3]	< 0.00 1
Work autonomy (reference, low autonomy)						
High autonomy				2.37 1	[1.87 2, 2.99 8]	< 0.00 1
Monthly income (reference, < 3 000)						
3 000–3 999				7.05 0	[1.39 9, 35.52 8]	0.01 8
4 000–4 999				3.29 7	[2.40 8, 4.51 0]	< 0.00 1
≥ 5 000				1.44 1	[1.05 0, 1.98 0]	0.02 4
Hours worked per week (reference, < 40)						
40–56				4.43 6	[0.54 4, 36.15 3]	0.16 4
≥ 57				2.00 6	[0.16 4, 24.54 0]	0.58 6
Nurses with expanded roles						
Proportion of working time on public health (reference, < 20)						
20–40	3.93 8	[0.42 4, 36.59 8]	0.22 8	3.94 2	[0.41 7, 37.21 1]	0.23 1
40–60	0.41 6	[0.10 0, 1.72 3]	0.22 6	0.38 9	[0.10 0, 1.51 5]	0.17 3
60–80	0.10 4	[0.02 4, 0.45 2]	0.00 3	0.16 0	[0.03 7, 0.69 4]	0.01 4
80–100	0.02 2	[0.00 3, 0.15 2]	< 0.00 1	0.04 4	[0.00 6, 0.31 9]	0.00 2
Work autonomy (reference, low autonomy)						
High autonomy				4.06 4	[1.30 1, 12.69 6]	0.01 6
Monthly income (reference, < 3 000)						
3 000–3 999				8.93 1	[1.12 9, 70.64 7]	0.03 8
4 000–4 999				4.69 0	[1.22 9, 17.89 8]	0.02 4
≥ 5 000				4.81 5	[1.16 9, 19.82 3]	0.03 0
Hours worked per week (reference, < 40)						
40–56				0.68 2	[0.02 2, 21.26 1]	0.82 8
≥ 57				1.10 3	[0.02 4, 51.40 6]	0.96 0

Work autonomy was measured by decision-making autonomy, which was surveyed by asking “Have your opinions and suggestions be considered and referenced regarding decisions on institutional development?” (Yes or no)

## Abbreviations

CI: Confidence intervals
CNY: Chinese Yuan
EBPH: Equalization of basic public health services
ORs: Odds ratios
PCPs: Primary healthcare providers
PHC: Primary health care
PHWs: Public health workers
SES: Social-economics status
THCs: Township healthcare centers

## References

[1] Topp S M, Abimbola S, Joshi R, et al. How to assess and prepare health systems in low-and middle-income countries for integration of services—a systematic review. Health Policy Plan 2017; 33(2): 298-312. doi: 10.1093/heapol/czx169 PMID: 29272396 10.1093/heapol/czx169 PMC5886169 29272396

[2] Zakumumpa H, Rujumba J, Kwiringira J, et al. Understanding the persistence of vertical (stand-alone) HIV clinics in the health system in Uganda: a qualitative synthesis of patient and provider perspectives. BMC Health Serv Res 2018; 18(1): 690. doi: 10.1186/s12913-018-3500-4 PMID: 30185191 10.1186/s12913-018-3500-4 PMC6126041 30185191

[3] Policy Research Department, Ministry of Health. Brigade health facilities could be contract to barefoot doctors. Health News. Jan 6, 1983 (accessed Nov 16, 2017). Chinese. Available from:http://ex.cssn.cn/ddzg/ddzg_ldjs/ddzg_sh/201701/t20170110_3377737_2.shtml

[4] Hu S, Tang S, Liu Y, Zhao Y, Escobar ML, de Ferranti D. Reform of how health care is paid for in China: challenges and opportunities. Lancet 2008;372(9652):1846-1853. doi: 10.1016/s0140-6736(08)61368-9 PMID: 18930520 10.1016/S0140-6736(08)61368-9 18930520

[5] Tang S, Meng Q, Chen L, Bekedam H, Evans T, Whitehead M. Tackling the challenges to health equity in China. Lancet 2008;372(9648):1493-1501. doi: 10.1016/s0140-6736(08)61364-1 PMID: 18930531 10.1016/S0140-6736(08)61364-1 PMC7135088 18930531

[6] The Zero Mark-up Policy for essential medicines at primary level facilities China case study. Geneva: World Health Organization; 2015. Available from: http://apps.who.int/iris/bitstream/handle/10665/188623/WHO_HIS_HGF_CaseStudy_15.2_eng.pdf;jsessionid=5440A9ECD0771049701D5618FA075B6B?sequence=1

[7] Yip WC, Hsiao WC, Chen W, Hu S, Ma J, Maynard A (2012). Early appraisal of China’s huge and complex health-care reforms. Lancet.

[8] Yip WC, Hsiao W, Meng Q, Chen W, Sun X. Realignment of incentives for health-care providers in China. Lancet 2010;375:1120-1130. doi: 10.1016/S0140-6736(10)60063-3 PMID: 20346818 10.1016/S0140-6736(10)60063-3 20346818

[9] Chen Z. Launch of the health-care reform plan in China. Lancet 2009;373 (9672):1322–1324. doi: 10.1016/S0140-6736(09)60753-4 PMID: 19376436 10.1016/S0140-6736(09)60753-4 19376436

[10] People's Republic of China health system review. Geneva: World Health Organization; 2015. Available from: http://iris.wpro.who.int/bitstream/handle/10665.1/11408/9789290617280_eng.pdf;sequence=1

[11] Coomber B, Barriball KL. Impact of job satisfaction components on intent to leave and turnover for hospital-based nurses: a review of the research literature. Int J Nurs Stud 2007;44(2):297-314. doi: 10.1016/j.ijnurstu.2006.02.004 PMID: 16631760 10.1016/j.ijnurstu.2006.02.004 16631760

[12] Rowe AK, de Savigny D, Lanata CF, Victora CG. How can we achieve and maintain high-quality performance of health workers in low-resource settings? Lancet 2005;366(9490):1026-1035. doi: 10.1016/s0140-6736(05)67028-6 PMID: 16168785 10.1016/S0140-6736(05)67028-6 16168785

[13] AbuAlRub RF. Job stress, job performance, and social support among hospital nurses. J Nurs Scholarsh. 2004;36(1):73-78. doi: PMID: 15098422 10.1111/j.1547-5069.2004.04016.x 15098422

[14] Pfeffer J. Building sustainable organizations: the human factor. Acad Manag Perspect 2010;24(1):34-45. Available from: https://www.researchgate.net/profile/Jeffrey_Pfeffer/publication/46479660_Building_Sustainable_Organizations_The_Human_Factor/links/56cbb52f08ae96cdd06fd53a/Building-Sustainable-Organizations-The-Human-Factor.pdf?origin=publication_detail

[15] Li L, Hu H, Zhou H, He C, Fan L, Liu X, et al. Work stress, work motivation and their effects on job satisfaction in community health workers: a cross-sectional survey in China. BMJ Open 2014;4(6):e004897. doi: 10.1136/bmjopen-2014-004897 PMID: 24902730 10.1136/bmjopen-2014-004897 PMC4054641 24902730

[16] Ge C, Fu J, Chang Y, Wang L. Factors associated with job satisfaction among Chinese community health workers: a cross-sectional study. BMC Public Health 2011;11:884. doi: 10.1186/1471-2458-11-884 PMID: 22111511 10.1186/1471-2458-11-884 PMC3248884 22111511

[17] Shi L, Song K, Rane S, Sun X, Li H, Meng Q. Factors associated with job satisfaction by Chinese primary care providers. Prim Health Care Res Dev 2014;15(1):46-57. doi: 10.1017/S1463423612000692 PMID: 23388523 10.1017/S1463423612000692 23388523

[18] Lu Y, Hu XM, Huang XL, Zhuang XD, Guo P, Feng LF, et al. Job satisfaction and associated factors among healthcare staff: a cross-sectional study in Guangdong Province, China. BMJ Open 2016;6(7):e011388. doi: 10.1136/bmjopen-2016-011388 PMID: 27436667 10.1136/bmjopen-2016-011388 PMC4964254 27436667

[19] Li L, Zhang Z, Sun Z, Zhou H, Liu X, Li H, et al. Relationships between actual and desired workplace characteristics and job satisfaction for community health workers in China: a cross-sectional study. BMC Fam Pract 2014;15:180-190. doi: 10.1186/s12875-014-0180-y PMID: 25403924 10.1186/s12875-014-0180-y PMC4240823 25403924

[20] Fang P, Luo Z, Fang Z. What is the job satisfaction and active participation of medical staff in public hospital reform: a study in Hubei province of China. Hum Resour Health 2015;13:34. doi: 10.1186/s12960-015-0026-2 PMID: 25975721 10.1186/s12960-015-0026-2 PMC4449964 25975721

[21] Hung LM, Shi L, Wang H, Nie X, Meng Q. Chinese primary care providers and motivating factors on performance. Fam Pract 2013;30(5):576-586. doi: 10.1093/fampra/cmt026 PMID: 23788201 10.1093/fampra/cmt026 23788201

[22] Landon BE. Career satisfaction among physicians. JAMA 2004;291(5):634. doi: 10.1001/jama.291.5.634 PMID: 14762046 10.1001/jama.291.5.634 14762046

[23] Stoddard Jeffrey J., Hargraves J. Lee, Reed Marie, Vratil Alison (2001). Managed Care, Professional Autonomy, and Income. Effects on Physician Career Satisfaction. Journal of General Internal Medicine.

[24] Linzer Mark, Konrad Thomas R., Douglas Jeffrey, McMurray Julia E., Pathman Donald E., Williams Eric S., Schwartz Mark D., Gerrity Martha, Scheckler William, Bigby JudyAnn, Rhodes Elnora (2000). Managed care, time pressure, and physician job satisfaction: Results from the physician worklife study. Journal of General Internal Medicine.

[25] Liu Q, Tian X, Tian J, Zhang X. Evaluation of the effects of comprehensive reform on primary healthcare institutions in Anhui Province*.* BMC Health Serv Res 2014;14: 268. doi: 10.1186/1472-6963-14-268 PMID: 24942901 10.1186/1472-6963-14-268 PMC4105389 24942901

[26] Zhao Y, Cui S, Yang J, Wang W, Guo A, Liu Y, et al. Basic public health services delivered in an urban community: a qualitative study. Public Health 2011; 125(1): 37-45. doi: 10.1016/j.puhe.2010.09.003 PMID: 21145087 10.1016/j.puhe.2010.09.003 PMC7118740 21145087

[27] Zhou H, Zhang W, Zhang S, Wang F, Zhong Y, Gu L, et al. Health providers’ perspectives on delivering public health services under the contract service policy in rural China: evidence from Xinjian County. BMC Health Serv Res 2015; 15: 75. doi: 10.1186/s12913-015-0739-x PMID: 25889866 10.1186/s12913-015-0739-x PMC4348399 25889866

[28] National Health and Family Planning Commission. Health statistics yearbook 2017. Beijing: National Health and Family Planning Commission; 2017. Chinese. Available from: http://www.yearbookchina.com/navibooklist-N2017120225-1.html

[29] WARR PETER, COOK JOHN, WALL TOBY (1979). Scales for the measurement of some work attitudes and aspects of psychological well-being. Journal of Occupational Psychology.

[30] Konrad Thomas R., Williams Eric S., Linzer Mark, McMurray Julia, Pathman Donald E., Gerrity Martha, Schwartz Mark D., Scheckler William E., Van Kirk Judith, Rhodes Elnora, Douglas Jeff (1999). Measuring Physician Job Satisfaction in a Changing Workplace and a Challenging Environment. Medical Care.

[31] Scheurer D, McKean S, Miller J, Wetterneck T. U.S. physician satisfaction: a systematic review. J Hosp Med 2009;4(9):560-568. doi: 10.1002/jhm.496 PMID: 20013859 10.1002/jhm.496 20013859

[32] Franco Lynne Miller, Bennett Sara, Kanfer Ruth (2002). Health sector reform and public sector health worker motivation: a conceptual framework. Social Science & Medicine.

[33] Li T, Lei T, Sun F, Xie Z. Determinants of village doctors’ job satisfaction under China’s health sector reform: a cross-sectional mixed methods study. Int J Equity Health 2017;16(1):64. doi: 10.1186/s12939-017-0560-8 PMID: 28420396 10.1186/s12939-017-0560-8 PMC5395962 28420396

[34] Wang H, Tang C, Zhao S, Meng Q, Liu X. Job satisfaction among health-care staff in township health centers in rural China: results from a latent class analysis. Int J Environ Res Public Health 2017;14(10): E1101 doi: 10.3390/ijerph14101101 PMID: 28937609 10.3390/ijerph14101101 PMC5664602 28937609

[35] Landon Bruce E. (2003). Changes in Career Satisfaction Among Primary Care and Specialist Physicians, 1997-2001. JAMA.

[36] Xu T, Cheng L, Xu Y. The integration of clinical medicine and public health in China. Shandong: The 7th annual conference of shandong medical ethics society; 2010. Chinese. Available from: http://kns.cnki.net/KCMS/detail/detail.aspx?dbcode=CPFD&dbname=CPFDLAST2017&filename=SDYL201010001027&v=MTI3MTVVS3JpZlp1NXVGQ250VXI3S0pWb2NOaW5TWXJHNEg5SE5yNDlGWmVzTkN4Tkt1aGRobmo5OFRuanFxeGRFZU1P

[37] Abera M, Tesfaye M, Belachew T, Hanlon C. Perceived challenges and opportunities arising from integration of mental health into primary care: a cross-sectional survey of primary health care workers in south-west Ethiopia. BMC Health Serv Res 2014; 14: 113. doi: 10.1186/1472-6963-14-113. PMID: 24602215 10.1186/1472-6963-14-113 PMC3946769 24602215

[38] Sweeney S, Obure CD, Terris-Prestholt F., et al. The impact of HIV/SRH service integration on workload: analysis from the Integra Initiative in two African settings. Hum Resour Health 2014; 12(1): 42. doi: 10.1186/1478-4491-12-42 PMID: 25103923 10.1186/1478-4491-12-42 PMC4130428 25103923

[39] Nsona H, Mtimuni A, Daelmans B, et al. Scaling up integrated community case management of childhood illness: update from Malawi. Am J Trop Med Hyg 2012; 87(5): 54-60. doi: 10.4269/ajtmh.2012.11-0759 PMID: 23136278 10.4269/ajtmh.2012.11-0759 PMC3748522 23136278

